# Supplementary material for: Risk score models for urinary tract infection hospitalization
Source: PLoS One. 2024 Jun 14;19(6):e0290215. doi: 10.1371/journal.pone.0290215 (PMC11178184; doi:10.1371/journal.pone.0290215)
Supplement: S1 Table — (DOCX) [file pone.0290215.s001.docx]

# **Supporting Information**

| Criteria | Score | Pos. Patient Score | Neg. Patient Score |
| --- | --- | --- | --- |
| Was the patient diagnosed with cognitive disorders, such as dementia, delirium? | 1 | 0 | 0 |
| Was the patient admitted to CCU in the past three months? | 2 | 0 | 0 |
| Was the patient admitted to ICU in the past three months? | 2 | 0 | 0 |
| Did the patient have low inpatient comorbidities in the past month? | -3 | 0 | 0 |
| Did the patient have low SNF comorbidities in the past month? | -1 | -1 | -1 |
| Was the total inpatient and SNF costs of the patient in the past six months low? | 1 | 1 | 0 |
| **Total Score (threshold = -1, where score that is greater predicts event)** |  | 0 | -1 |
| **Prediction** |  | 1 | 0 |

S1 Table. Integerized LR scoring example. The method obtains the correct prediction outcome for these two patients*.*
