# Supplementary material for: Risk score models for urinary tract infection hospitalization
Source: PLoS One. 2024 Jun 14;19(6):e0290215. doi: 10.1371/journal.pone.0290215 (PMC11178184; doi:10.1371/journal.pone.0290215)
Supplement: S2 Table — (DOCX) [file pone.0290215.s002.docx]

# **Supporting Information**

| Criteria | Pos. Patient category | Pos. Patient Score | Neg. Patient category | Neg. Patient score |
| --- | --- | --- | --- | --- |
| Age | 4 | -1 | 5 | 0 |
| Elixhauser inpatient score | 2 | -2 | 2 | -2 |
| Elixhauser SNF score | 1 | -2 | 1 | -2 |
| Delirium, dementia, amnestic and other cognitive disorders | 0 | -3 | 0 | -3 |
| UTI infection diagnosis more than 6 months | 1 | 1 | 1 | 1 |
| Bacterial infection diagnosis more than 6 months | 0 | -1 | 0 | -1 |
| ICU within last 3 months | 0 | -1 | 0 | -1 |
| Rate of readmission within 30 days after discharge from hospital | 3 | 1 | 3 | 1 |
| Carrier cost within 6 months | 3 | 2 | 5 | -2 |
| Total inpatient and outpatient cost during last 6 months | 1 | 7 | 4 | -3 |
| **Total Score (threshold = -6, where score that is greater predicts event)** |  | 1 |  | -12 |
| **Prediction** |  | 1 |  | 0 |

S2 Table. Credit Scorecard scoring example. The method obtains the correct prediction outcome for these two patients.
